# Momentary predictors of dissociation in functional neurological disorder: an ecological momentary assessment-based pilot study

**DOI:** 10.3389/fpsyt.2026.1798482

**Published:** 2026-04-30

**Authors:** Amy Crellin, L. S. Merritt Millman, Matthew Hotopf, Susannah Pick

**Affiliations:** Department of Psychological Medicine, Institute of Psychiatry, Psychology & Neuroscience, King’s College London, London, United Kingdom

**Keywords:** Depersonalisation, derealisation, dissociation, dissociative disorder, functional motor disorder, functional neurological disorder, functional seizures, somatoform disorder

## Abstract

**Introduction:**

Evidence suggests that dissociation may play a role in the manifestation of functional neurological disorder (FND). Dissociative experiences are frequently reported in FND, yet their dynamic associations with affective and physiological states remain underexplored. This pilot study aimed to examine dissociative symptoms in daily life in FND, to identify predictors of dissociation using ecological momentary assessment (EMA) combined with wearable heart-rate monitoring.

**Methods:**

Seventeen individuals with FND (functional seizures/motor symptoms) and seventeen age- and gender-matched healthy controls completed EMA via pseudorandom smartphone prompts eight times daily for one week. This was a secondary analysis of data from a pilot case-control intensive longitudinal observation study. Dissociation (depersonalisation, derealisation, amnesia), negative affect, and subjective arousal were assessed using items modified from validated scales, while heart-rate was continuously recorded via Fitbit devices. Multilevel modelling was conducted to examine between-group differences, and momentary associations between predictors (negative affect, subjective arousal, heart-rate) and dissociative symptoms within the FND group. Time-lagged analyses explored temporal relationships between the predictors and dissociative symptoms.

**Results:**

The FND group reported significantly higher dissociative symptoms across the week compared to controls; amnesia (F(1,34)=13.09, *p* <.001), depersonalisation (F(1, 34)=7.35, *p* = .010), and derealisation (F(1, 34)=8.31, *p* = .007), these differences withstood correction for anxiety, but not for depression. Negative affect (*p-values*=<0.001, β=0.094-0.111), subjective arousal (*p-values*=<0.001, β=0.102-0.124), and higher heart-rate (*p-values* = 0.01-0.006, β= 0.078-0.091) were each significant concurrent predictors of all three dissociative symptom types. In combined models, negative affect and subjective arousal remained robust predictors across all symptom domains, whereas heart-rate lost significance. Time-lagged analyses did not yield significant associations.

**Conclusion:**

Individuals with FND self-reported elevated dissociative symptoms in daily life compared to healthy controls; however, these significant differences did not remain when controlling for depression. Dissociation was consistently associated with subjective arousal and negative affect, but not heart-rate, underscoring the importance of subjective emotional states over physiological influences on self-reported dissociative symptom variability. The absence of temporal effects highlights the transient nature of dissociation in FND. These findings support the possibility of conceptualising FND through a dissociative lens and emphasise the need for larger longitudinal studies to clarify underlying mechanisms.

## 1 Introduction

Functional neurological disorder (FND) is a common disorder, with an approximate prevalence of 50 for every 100,000 of the population (1). The disorder can be highly disabling (2) and is often associated with poor clinical outcomes (3). FND can give rise to alterations in motor skills/function, cognition and sensory processing, which usually leads to impairment and/or distress (4). Whilst there has been a gravitation towards recognising the neuroscientific foundations underpinning FND (5), it is also acknowledged that FND symptoms might be mediated by dissociation (6–9).

Dissociation as a symptom is characterised by feelings of disconnection from oneself (10), others and one’s surroundings, or the segregation of more complex cognitive and behavioral processes such as memory, language or planning (11). During dissociative episodes, there can be a disruption in the ability to integrate memories, consciousness and identity (12). There is a current discrepancy between the International Classification of Diseases 11^th^ Edition (ICD-11) and the Diagnostic and Statistical Manual of Mental Disorders 5^th^ Edition (DSM-5) in relation to the categorisation of FND. The DSM-5 characterises FND as a somatic symptom disorder (13), whereas the ICD-11 refers to FND as “dissociative neurological symptom disorder” (14). Increasingly, empirical studies have found evidence to support dissociative phenomena as foundational processes which may play a role in a range of FND symptoms (15, 16).

Meta-analytic evidence shows that psychological dissociative symptoms, such as depersonalisation, derealisation or amnesia, are significantly higher in FND groups relative to neurological or healthy controls (16). Additionally, comorbidities between FND and other dissociative disorders are prevalent (17, 18). Positive associations between dissociative symptom scores and FND symptom frequency/severity have been found by a number of researchers (7, 19–22), and many patients with functional seizures report dissociative symptoms prior to and/or during the events (Pick, Mellers & Goldstein, 2016; 7, 23). Finally, in a study which examined directly the influence of dissociative experiences on interoceptive (bodily) awareness in individuals with FND, the FND group exhibited higher baseline state (momentary) dissociation (8). After a dissociative episode was experimentally induced with a validated procedure (24, 25), interoception, which had previously been intact at baseline, became impaired, with higher dissociation scores inversely correlated with interoceptive accuracy (8).

Dissociative experiences, particularly depersonalisation and derealisation, are often reported by individuals with FND, and are thought to reflect involuntary disruptions in emotional and autonomic regulation, potentially manifesting in hypo- or hyper-aroused states (26). Alterations in indices of physiological/subjective arousal and affect may therefore be expected to occur prior to, or alongside, dissociative experiences. Elevated heart-rate, for example, has been associated with autonomic dysregulation in FND (27), with evidence also suggesting that altered cardiac processing (how the brain receives and responds to signals from the heart) may contribute to dissociative symptoms (28). Negative affect, including heightened anxiety and distress, is commonly observed in FND and may overwhelm emotional regulation systems, leading to dissociation as a coping response (7, Lanius et al., 2010). Similarly, high levels of subjective arousal have been associated with fluctuations in dissociative states in a clinical population with a diagnosis of borderline personality disorder, particularly in emotionally charged contexts (29).

The present study is a secondary analysis of data from an existing pilot study into biopsychosocial antecedents and correlates of FND symptoms (39), which preceded a larger-scale investigation (30). In the present study, data captured through ecological momentary assessment (EMA) and wearable technology was combined to ascertain potential associations between dissociative experiences and FND symptoms. The use of EMA is a strength when examining the variability of dissociation, given that dissociative experiences are often brief and transient, or have a sudden onset in response to external or stressful stimuli (31). Sampling dissociative experiences multiple times daily, as in EMA, allows for deeper insights into the precursors and correlates of dissociation across timepoints. This approach extends findings derived from traditional outcome measures of dissociation, which usually ask participants to aggregate their ratings by recalling dissociative experiences from the last month/fortnight (32).

Here, we report differences between dissociation levels in a group with FND in comparison to healthy controls, and a secondary analysis of EMA data, which aimed to examine the severity and possible predictors/correlates of dissociative symptoms in the FND group. We focused on three possible predictors of relevance to the potential divide between subjective experience and objective measures in affect and arousal states - negative affect, subjective arousal, and objective arousal (i.e., heart-rate). Outcome variables were three common types of dissociative experience - amnesia, depersonalisation and derealisation. These data were obtained in 2022 as part of the broader study mentioned above by Pick et al. (39).

The objectives were to test several hypotheses grounded in theory and evidence:

1. Compared to healthy controls, participants with FND were predicted to have significantly higher aggregated scores across the week-long sampling period, for all three dissociative symptom types: dissociative amnesia, depersonalisation and derealisation (7, 16).
2. Momentary amnesia, depersonalisation and derealisation scores would correlate with, and/or be temporally predicted by, negative affect, subjective physiological arousal and heart-rate (27, 30, 33, 34).

## 2 Materials and methods

Ethical approval was obtained from King’s College London High Risk Research Ethics Committee (HR/DP-21/22-28,714). Co-production of the study design was ensured through input from an FND Patient and Carer Advisory panel. All data were collected by SP from July-October 2022.

### 2.1 Participants

Seventeen healthy controls were recruited alongside 17 people with a primary diagnosis of FND. Participants with FND were recruited through two charities (FND Hope, FND Action), both of which provide support and resources to those with the diagnosis. Healthy controls were sought via social media advertisements and matched to participants in the FND group by age and gender, at group level. All participants needed to have normal or corrected vision, to speak English fluently and be between 18–65 years of age.

Inclusion criteria for the FND group were having a primary diagnosis of FND, with seizures and/or motor symptoms, which was confirmed by the participants providing a document from a clinician that attested to their diagnosis. Participants with seizures as a primary symptom were required to experience a baseline rate of two or more episodes per month, to allow for adequate data collection. Exclusion criteria for the FND group were physical or cognitive symptoms or a disability that would inhibit their ability to complete any aspect of the broader study, such as upper limb paralysis or constant tremor, in addition to comorbidities encompassing significant cardiovascular, psychiatric or neurological conditions (such as epilepsy, or Parkinson’s disease). Exclusion of these disorders was applied to maximise engagement and completion rates, and to reduce the chance of confounding the analyses. Candidates were excluded from the healthy control group if they had a current or historical diagnosis of FND and/or if they were experiencing any current major physical or mental health condition.

This study was originally designed to establish the feasibility and acceptability of the data collection methods used, and as such, an a-priori power analysis was not conducted, and recruitment numbers were based on practical factors rather than an adequately powered sample size.

### 2.2 Materials and measures

At baseline, participants completed brief cognitive and clinical assessments. Cognitive ability was measured using the Wechsler Abbreviated Scale of Intelligence-I Second Edition (WASI-II) (35) for verbal and nonverbal IQ, and selected Cambridge Neuropsychological Test Automated Battery (CANTAB) tasks assessing neurocognitive and psychomotor functions relevant to FND (Cambridge Connect, 2019), reported in Pick et al. (36). An abbreviated Structured Clinical Interview for DSM-5 Disorders - Research Version (SCID-5-RV) screened for exclusionary diagnoses (37). Self-report measures assessed FND symptoms, physical complaints, depression, anxiety, dissociation, alexithymia, autistic traits, trauma history, illness beliefs, daily functioning, and health-related quality of life, which are reported elsewhere (22, 38; Pick, 38; Pick, 39). The Generalised Anxiety Disorder-7 [GAD-7] (40) and the Patient Health Questionnaire [PHQ-9] (41) were also administered to assess for anxiety and depression scores respectively and these were used for sensitivity analyses.

On completion of an in-person laboratory testing session (30, 36, 38, 39), participants were provided with a wearable device that recorded their heart-rate. The Fitbit Charge 5 was chosen as it was deemed to be a discrete device with adequate monitoring available through photo-plethysmography. The Fitbit was worn on the non-dominant wrist, and participants were provided with an anonymised login to a Fitbit account. Participants were asked to sync the Fitbit daily to this account, which was managed by the research team, and to decline any prompts to sync the device with their personal devices. The Fitbit measured heart-rate continuously, with output in beats per minute. Each participant’s data was deleted from the Fitbit platform at the earliest opportunity and held securely in line with guidance from King’s College London, and within their secure servers.

The RealLife Exp app (LifeData, LLC) administered ecological momentary assessment (EMA) prompts to participants’ smartphones eight times per day, following a pseudorandom schedule between 8:00 a.m. and 10:00 p.m. across the course of one week. The schedule ensured a minimum interval of one hour between alerts (https://www.lifedatacorp.com/). There was an approximate average of one hour and 45 minutes between alerts. The prompts requested responses to questions related to primary FND symptoms and a range of potential predictors of these symptoms, detailed in full in Pick et al. (2024). The dissociation items included momentary assessments of amnesia, depersonalisation, and derealisation. Subjective negative affect and physiological arousal ratings were also obtained at each prompt. The affect questions were adapted from the Positive and Negative Affect Schedule (PANAS) (42) and the dissociation items from the Clinician-Administered Dissociative States Scale (CADSS) (43).

To reduce the burden placed on participants, only a few items were selected from each scale. The items selected for use in this study were those with the strongest factor loadings. As indicated in Bremner et al. (43) and Watson et al. (42), both scales have shown high internal consistency, validity, and reliability. Cronbach’s alpha was calculated for the negative affect items (α=.78), depersonalisation (α=.90), derealisation (α=.81), amnesia (α=.64), and total dissociation items (α=.91) that were included in EMA prompts in this study. Variance inflation factors (VIF) were examined to assess multicollinearity between negative affect and subjective arousal. The VIF values were low (1.18), suggestive of no evidence of problematic multicollinearity. All questions posed to participants were scored using a 7-point Likert scale (**Table 1**).

**Table 1:** Questions and scoring from ecological momentary assessment questionnaire as presented in *Pick et al. (2024)* license CC-BY4.

Domain	Question and scale for scoring (1 = Not at all – 7 = Extremely):
Dissociation: Dissociative amnesia	a) I cannot account for things that have recently happened. b) I feel spaced out, and/or have lost track of what is going on.
Dissociation: Depersonalisation	a) I feel disconnected from my own body. b) I feel separated from what is happening to me, like an actor in a movie, or a robot.
Dissociation: Derealisation	a) Things seem unreal to me, as if I am in a dream. b) It seems like I am looking at the world through a fog.
Negative affect	I feel: - Scared - Upset - Nervous - Ashamed - Irritable - Hostile
Arousal	I feel bodily arousal. *

*Participants were instructed to report on bodily arousal reflecting markers of sympathetic/autonomic arousal, with examples provided (e.g. racing heart, sweating, dry mouth).

### 2.3 Procedures

All participants gave written informed consent prior to data collection, in line with the Declaration of Helsinki. Training and support were provided to download and understand the app used for the collation of data, and the Fitbit Charge 5. Participants were asked to complete an initial session with the RealLife Exp app, with data collection commencing the day after. Participants were offered a remote debrief session after they had completed the EMA data collection and were provided a £50 shopping voucher to compensate for their time and effort in completing the EMA protocol.

### 2.4 Data processing and statistical analysis

There was a maximum of 952 datapoints per group, and the completion rate was >80%. For the purpose of the primary analysis related to between-group week-level differences, data were aggregated at week-level for each participant. Data were cleaned and missing values were imputed with the nearest value that was last recorded within the same day, when establishing aggregated scores. Heart-rate data were missing for 26% of EMA prompts for the FND group and 28% of the healthy control group, primarily due to reasons including device loss, technical issues, or non-wearing by choice apparently unrelated to symptoms, hence were missing completely at random (MCAR). Multilevel models were estimated using maximum likelihood, which uses all available observations under a missing-at-random assumption. Multilevel modelling can accommodate incomplete data under maximum likelihood estimation (44), and as such we did not apply a predefined missing-data exclusion threshold, nor were missing values imputed, following the approach taken by Pick et al. (39) in the original manuscript. To control for the risk of false discoveries due to multiple comparisons, the Benjamini-Hochberg correction was applied to adjust alpha levels and maintain the false discovery rate at an acceptable threshold of 5%.

Statistical analyses were run using SPSS version 28 for Mac. The results reported herein have not been previously published and have a different focus to that of the primary analyses reported by Pick et al. (39). Multilevel (linear mixed effects) modelling was used to examine the data across two levels (45). The first level was the moment-to-moment scores within-participants for negative affect, levels of subjective arousal and heart-rate in conjunction with dissociative symptom domain scores (27, 30, 33, 34). The second level were the individual participants. This allowed for variance both within-participants and between-participants to be modelled.

Week-level between-group differences in dissociation scores were calculated using multilevel models, with group (FND, HC) entered as a fixed effect, and with participant entered as a random effect. These analyses were then rerun controlling for possible confounding effects of anxiety and depression, using the GAD-7 and PHQ-9.

Arousal, negative affect and heart-rate were included as fixed effects in separate models to estimate their influence on dissociative symptoms across all participants. The predictors were person-mean centred to isolate within-person effects. This approach separated momentary deviations from a participant’s typical level, from stable between-person differences. Consequently, the model coefficients reflected whether increases in a predictor relative to a participant’s own mean were associated with concurrent changes in dissociative symptoms. This enabled examination of population-level associations while accounting for individual differences through participant-level random intercepts (45). Initially, multilevel models were run examining predictors separately. Significant predictors were then included in a multi-predictor multilevel model. Unconditional multilevel models were used to calculate intraclass correlation coefficients (ICCs) (45). These indicated substantial clustering at the participant level across outcomes. For amnesia, the ICC was .57, indicating that 57% of the variance occurred between individuals and 43% within individuals. For depersonalisation, the ICC was .79, with 79% of variance attributable to between-person differences. For derealisation, the ICC was .81, indicating that 81% of variance occurred between individuals and 19% reflected within-person fluctuation. These findings demonstrate substantial non-independence of observations and support the use of multilevel modelling.

Following the approach taken for FND symptoms in Pick et al. (2024), analyses were run using concurrent and time-lagged predictor values. Scores for predictor variables at a given timepoint were examined in relation to dissociation scores at the same (concurrent) or next (time-lagged) timepoint. These analyses were run separately in the FND group and healthy controls. Scores for the GAD-7 and PHQ-9 were entered as covariates in sensitivity analyses; however, attempts to control for use of medication, mental health conditions and physical health conditions were not feasible. These variables in conjunction were highly correlated, clustered within the same individuals and were largely absent from the HC group by definition. When multiple overlapping level 2 predictors were entered simultaneously, the model was unable to partition their independent effects, which meant it was not statistically possible to control for these variables concurrently. To retain model stability and interpretability, a parsimonious approach was maintained and did not include all possible confounders.

## 3 Results

Seventeen participants with FND were recruited: five with a diagnosis of functional seizures and twelve with functional movement disorder. These participants were frequency matched for gender and age with seventeen healthy controls. The employment rate was higher in the healthy control group, and comorbid mental and physical health diagnoses were more common in the FND group (**Table 2**).

**Table 2:** Participant demographics adapted from *Pick et al. (2024)* license: CCBY4.

Demographic:	FND group n (%)	Healthy controls n (%)	Statistical values
Age (years):	36.5 (SD = 10.6)	39 (SD = 11)	t(32)=0.67, *p* = 0.51
Relationship status: (%) married or living with a partner	10 (59)	8 (47)	χ^2^ = 12, *p* = 0.73
Mental health diagnosis (% yes)	10 (59)	1 (6)	*p* = 0.001*
Physical health diagnosis (% yes)	12 (71)	4 (24)	*p* = 0.007*
Ethnicity (% white)	14 (82)	12 (71)	*p* = 0.688^*^
Employment (% in employment)	2 (12)	13 (76)	*p* < 0.001*
Education (% post-age of 16 years)	16 (94)	17 (100)	*p* = 1.00*
Gender (% female)	13 (76)	13 (76)	*p* = 0.656*
Medication (% currently taking medication)	16 (94)	5 (29)	*p* < 0.001*

*Fishers exact.

### 3.2 Week-level between-group differences

Unadjusted multilevel models demonstrated significant between-group differences across all dissociative outcomes, with FND participants reporting higher amnesia (F(1,34)=13.09, *p* <.001), depersonalisation (F(1, 34)=7.35, *p* = .010), and derealisation (F(1, 34)=8.31, *p* = .007) than healthy controls.

Adjustment for anxiety (GAD-7) did not eliminate group differences, and GAD-7 was not a significant predictor of dissociative outcomes. In contrast, adjustment for depressive symptoms (PHQ-9) attenuated all group effects, particularly for amnesia, where PHQ-9, rather than group was a significant predictor. However, depressive symptoms did not significantly predict depersonalisation or derealisation (**Table 3**).

**Table 3:** Between-group differences, including when controlled for effects of anxiety (GAD-7) & depression (PHQ-9) on dissociative outcomes.

Outcome	Predictor	F	df	p
Amnesia	Group	8.60	1, 41.11	.005
	GAD-7	1.94	1, 240.28	.165
Depersonalisation	Group	4.86	1, 38.32	.033
	GAD-7	3.44	1, 791.51	.064
Derealisation	Group	5.61	1, 37.89	.023
	GAD-7	3.13	1, 903.10	.077
Amnesia	Group	3.51	1, 52.87	.067
	PHQ-9	4.88	1, 173.95	.028
Depersonalisation	Group	3.45	1, 47.54	.070
	PHQ-9	2.20	1, 670.55	.138
Derealisation	Group	3.86	1, 46.66	.055
	PHQ-9	2.85	1, 747.47	.092

GAD-7 (Generalised Anxiety Disorder 7-item scale) is a short self-report measure of anxiety symptom severity, and PHQ-9 (Patient Health Questionnaire 9-item scale) is a brief self-report measure of depressive symptom severity.

### 3.3 Momentary predictors of dissociative symptoms

The adjusted ICC for the three dissociative symptom domains were: amnesia (0.534); depersonalisation (0.633); and derealisation (0.606). These results confirmed that multilevel modelling was appropriate and suggested that, across all outcome variables, over half of the variance between symptoms scores was due to stable between-participant differences. In the concurrent models wherein predictors were examined separately, negative affect, heart-rate and subjective arousal were all significantly correlated with the three dissociative symptoms in the FND group (**Table 4**). These results withstood Benjamini-Hochberg correction.

**Table 4:** Results from concurrent multilevel modelling for the FND group per outcome variables when controlled for GAD-7 and PHQ-9 scores.

Outcome	Predictor	B	SE	95% CI	F	*p* value	Adjusted *p* value
Dissociative Amnesia	Arousal	0.102	0.026	[0.051, 0.153]	15.86	<.001	.005
	Negative Affect	0.094	0.027	[0.041, 0.147]	12.30	<.001	.011
	Heart-rate	0.078	0.030	[0.019, 0.137]	6.62	.010	.016
Depersonalisation	Arousal	0.114	0.031	[0.053, 0.175]	13.41	<.001	.022
	Negative Affect	0.106	0.030	[0.047, 0.165]	12.64	<.001	.027
	Heart-rate	0.089	0.032	[0.026, 0.152]	7.76	.006	.033
Derealisation	Arousal	0.124	0.030	[0.065, 0.183]	17.17	<.001	.038
	Negative Affect	0.111	0.030	[0.052, 0.170]	13.69	<.001	.044
	Heart-rate	0.091	0.033	[0.027, 0.155]	7.65	.006	.05

In combined models, negative affect and subjective arousal significantly predicted dissociative symptoms, including amnesia (*p* = .001), depersonalisation (*p* = .001), and derealisation (*p* = .001), regardless of whether anxiety (GAD-7) (40) or depression (PHQ-9) (41) was controlled for (**Table 4**). In contrast, heart-rate was not a significant predictor in any of the combined models. These analyses in the healthy control group yielded no significant results.

A final concurrent multilevel model was conducted for negative affect and subjective arousal (**Table 5**) in the FND group. Across all dissociative symptom domains, negative affect and arousal maintained significance in the combined model. Negative affect and arousal were positively associated with amnesia, depersonalisation and derealisation.

**Table 5:** Results from multi-predictor multilevel modelling for the FND group.

Predictor:	Negative affect				
**Outcomes:**	*F*	*p*-value	*β*	*SE*	CI
Amnesia	17.404	<.001	0.230	0.055	[0.122, 0.338]
Depersonalisation	13.686	<.001	0.190	0.051	[0.089, 0.291]
Derealisation	24.375	<.001	0.235	0.048	[0.142, 0.329]
Predictor:	Arousal				
**Outcomes:**	*F*	*p*-value	*β*	*SE*	CI
Amnesia	24.141	<.001	0.121	0.025	[0.073, 0.170]
Depersonalisation	9.991	.002	0.073	0.023	[0.028, 0.118]
Derealisation	25.259	<.001	0.107	0.021	[0.065, 0.149]

Multilevel modelling was also conducted with the predictor variables time-lagged, which did not give rise to any significant results across both the FND and healthy control group.

## 4 Discussion

This analysis aimed to establish whether participants with FND differed from healthy controls in three common dissociative symptom types in everyday life - amnesia, depersonalisation and derealisation. We also aimed to explore real-time associations between subjective affect, arousal and dissociative symptoms in individuals with FND, on a moment-to-moment basis. The study used EMA to capture subjective experiences, combined with heart-rate data from a wearable device. Multilevel models conducted for week-level between-group differences, shown in **Table 3**, highlighted that the FND group experienced elevations in all three types of dissociation, compared to the healthy control group. These findings are consistent with previous studies, which have found evidence of greater dissociation in FND samples compared to controls using traditional retrospective self-report measures (7, 16, 23). These group level differences remained when controlling for anxiety using GAD-7 scores; however, they were attenuated when controlling for depressive symptoms via PHQ-9 scores. Notably, this secondary analysis of pilot data was not powered for definitive hypothesis testing, and findings should be interpreted as preliminary. Observed group differences and within-person associations require replication in larger, adequately powered samples before mechanistic or conceptual conclusions can be drawn. This work has now been conducted within the broader research programme (Pick, David et al. (46), with results from the larger study soon to be available.

Across all three dissociative outcomes, arousal and negative affect emerged as consistent and significant positive correlates. This indicated that increases in subjective negative emotional and arousal states are associated with increases in dissociation. In contrast, heart-rate was not a significant predictor in a combined model with the subjective factors, although this may have been affected, in part, by missing data. Despite the associations between momentary scores for perceived levels of arousal and emotional states, time-lagged analyses examining dissociative symptoms at the timepoint prior to the concurrent measurement did not evidence any significant associations. This finding further indicates the potential importance of the use of EMA as a data collection method, highlighting that within this FND sample, momentary data were more strongly correlated with dissociative symptoms than those collected a period of time before the symptoms/outcome. Given that the prompts for data completion were at random across a period of 14-hours daily, it is possible that when the next prompt arrived symptoms that had arisen as a result of the predictor had dissipated or were superseded by what was happening for the participant in the present moment. As such, further exploration may be needed to better understand the correct time-lag required. It would also be advantageous to consider if shorter lags between EMA prompts may have given rise to different findings.

Together, these findings may offer support to existing evidence showing that people with FND experience consistently higher levels of dissociative symptoms than non-clinical control samples (16) and demonstrate that subjective negative emotional states and perceived arousal co-occur with dissociative symptoms in this population. A previous study found that negative valence and arousal ratings were predictive of later dissociative symptoms in a sample of participants with borderline personality disorder (BPD) (29). Temporal relationships between these affective variables and dissociative symptoms warrant further exploration to identify potential differences in the role dissociation holds in FND versus other psychiatric disorders, such as BPD or post-traumatic stress disorder (PTSD). In a recent study by Beutler-Traktoveno et al. (47) EMA was used to examine heart-rate changes related to dissociative episodes, in a population with PTSD. The study did not find support for the theory that hypoarousal leads to differences in heart-rate, and heart-rate did not differ from baseline during dissociative episodes. These findings are aligned with those here, in that heart-rate lost significance as a predictor when modelled with negative affect and subjective arousal, however these findings should be interpreted with caution due to the small sample and level of missing heart-rate data. The present analyses also did not link fluctuations in self-reported dissociative symptoms to changes in reported FND symptoms, which would be important to further establish these connections.

The present findings highlight the dynamic and emotion-linked nature of dissociation in FND, reinforcing the potential importance of internal affective experiences over purely physiological indicators in understanding symptom variability. This concept was also highlighted by Pick et al. (30) in a study which considered the disconnect between affect and arousal. Results concluded that perceived arousal or levels of subjective negative affect were not aligned with a significant difference in heart-rate (30). The same study did find, however, that autonomic variables (skin conductance, heart-rate) were associated with worse FND symptom ratings following highly arousing negative affective stimuli, evidencing that heart-rate changes were more closely associated with FND symptom severity. There is other evidence to suggest that people experiencing FND subjectively report more distressing, stressful or anxiety provoking life events and circumstances than that of the general population (7, 48–50).

Previous research has found that people with FND experience more significant subjective or autonomic/reflexive levels of arousal or response to salient emotions, however not usually simultaneously (51). In a study by Herrero et al. (52) it was concluded that there was evidence of a mind-body disconnect in a group of females with a diagnosis of functional seizures, wherein strong subjective emotional experiences were found to be associated with reduced autonomic reactions. This supports the notion of a lack of integration between consciousness, cognition, emotions and the body in this population (51, 53). An important caveat, however, noted by Adewusi et al. (34), is that reporting on subjective and objective measurements concurrently is challenging, due to the momentary capture of physiological data, that may be influenced by other factors such as hunger or tiredness. In the current study, the reports of higher levels of subjective arousal may not have been matched by significantly elevated heart-rate, however this cannot be confirmed from the analysis as the focus was in relation to whether the covariates were predictive of dissociation. In the paper by Pick et al. (39) this was analysed, and results supported that for the healthy control group, heart-rate was a significant predictor of arousal, but not for the FND group. These findings may be representative of the potential mind-body disconnect that underpins dissociative phenomena and is conjectured to underlie and maintain functional neurological symptoms (8, 16, 38).

### 4.1 Strengths and limitations

This pilot study was the first to use EMA to examine predictors of dissociative experiences in an FND sample. By capturing repeated, within-person reports on dissociation, affective states and physiological arousal, it was possible to examine how moment-to-moment changes in arousal, negative affect and heart-rate related to dissociative experiences. The results suggest that there is value in obtaining data on moment-to-moment, dynamic fluctuations in emotions and arousal alongside dissociative symptoms. Using data collected via EMA allows for greater insights into the day-to-day nuances of a person’s experience that may be lost when using conventional reflective outcome measures with broad data collection windows such as “in the last four weeks”. Notably, dissociative symptoms such as amnesia, depersonalisation and derealisation often occur transiently and may be influenced by emotional and physiological states (55). This may result in the loss of accurate reporting when considering symptoms over a longer period retrospectively.

Nevertheless, this study was conducted primarily to examine the feasibility and acceptability of the data collection methods prior to a larger scale study (Pick, David, et al., 2024), thus it was insufficiently powered to draw generalisable conclusions and an a-priori power analysis was not conducted. Despite this, the method of data collection (EMA), with 56 datapoints per participant, serves to increase the power of the statistical analyses. The groups were matched for gender, with just over 75% of each group being female, to reflect the female preponderance in FND (54). The two groups differed on psychiatric and physical health comorbidity and medication status; therefore, residual confounding is possible. Associations attributed to group status may partially reflect comorbid symptoms or pharmacologic effects on affect, dissociation, or autonomic activity. Notably, the reported physical health conditions were primarily mild and unrelated (e.g., hay fever, minor sporting injuries), and therefore unlikely to systematically influence dissociative symptoms. Mental health diagnoses were predominantly depression and anxiety, which were captured dimensionally by the PHQ-9 and GAD-7 scores included in sensitivity analyses.

There was a significant number of missing data points for heart-rate, therefore the analysis of these data should be interpreted with caution. Finally, the reporting period was only one week, which is unlikely to be representative of the course of dissociative symptoms over the longer-term trajectory in FND.

### 4.2 Conclusions and future directions

This pilot study demonstrates that three types of psychological dissociative symptoms, depersonalisation, derealisation, and amnesia, are elevated in FND in everyday life, compared to healthy controls, albeit that this significant elevation dissipated when controlling for depression. We also demonstrate that negative affect and subjective arousal are correlates of dissociative experiences in FND, at a momentary level in this small sample. These findings indicate the potential clinical importance of assessing dissociation in patients with FND routinely, and, if present, providing psychological support to better manage these experiences. This pilot work has informed a subsequent larger-scale study using EMA to collect similar data over a longer-timescale from a sufficiently powered FND sample, in addition to capturing dissociative, affective and arousal states within a functional magnetic resonance imaging study (30). These investigations will provide further insights into relationships between momentary dissociative, affective, and autonomic states and neural activity in distinct FND subgroups.

## Data Availability

The raw data supporting the conclusions of this article will be made available by the authors, without undue reservation.

## References

[1] Finkelstein SA Diamond C Carson A Stone J . Incidence and prevalence of functional neurological disorder: a systematic review. J Neurol Neurosurg Psychiatry. (2025) 96:383–95. doi: 10.1136/jnnp-2024-334767. PMID: 39663114 PMC12015090

[2] Bennett K Diamond C Hoeritzauer I Gardiner P McWhirter L Carson A . A practical review of functional neurological disorder (FND) for the general physician. Clin Med. (2021) 21:28–36. doi: 10.7861/clinmed.2020-0987. PMID: 33479065 PMC7850207

[3] Gelauff J Stone J Edwards M Carson A . The prognosis of functional (psychogenic) motor symptoms: a systematic review. J Neurol. Neurosurg Psychiatry. (2014) 85:220–6. doi: 10.1136/jnnp-2013-305321. PMID: 24029543

[4] Hallett M Aybek S Dworetzky BA McWhirter L Staab JP Stone J . Functional neurological disorder: new subtypes and shared mechanisms. Lancet Neurol. (2022) 21:537–50. doi: 10.1016/S1474-4422(21)00422-1. PMID: 35430029 PMC9107510

[5] Perez DL Edwards MJ Nielsen G Kozlowska K Hallett M LaFrance WC . Decade of progress in motor functional neurological disorder: continuing the momentum. J Neurol. Neurosurg Psychiatry. (2021) 92:668–77. doi: 10.1136/jnnp-2020-323953. PMID: 33722822 PMC8440656

[6] Stone J Burton C Carson A . Recognising and explaining functional neurological disorder. BMJ. (2020) 371:m3745. doi: 10.1136/bmj.m3745. PMID: 33087335

[7] Pick S Mellers JDC Goldstein LH . Dissociation in patients with dissociative seizures: relationships with trauma and seizure symptoms. Psychol Med. (2017) 47:1215–29. doi: 10.1017/S0033291716003093. PMID: 28065191

[8] Pick S Rojas-Aguiluz M Butler M Mulrenan H Nicholson TR Goldstein LH . Dissociation and interoception in functional neurological disorder. Cogn Neuropsychiatry. (2020) 25:294–311. doi: 10.1080/13546805.2020.1791061. PMID: 32635804

[9] Blanco SR Mitra S Howard CJ Sumich AL . Psychological trauma, mood and social isolation do not explain elevated dissociation in functional neurological disorder (FND). Pers Individ Dif. (2023) 202:111952. doi: 10.1016/j.paid.2022.111952. PMID: 41936479

[10] Brown RJ . Dissociation and functional neurologic disorders. Handb Clin Neurol. (2016) 139:85–94. doi: 10.1016/B978-0-12-801772-2.00008-4. PMID: 27719880

[11] Holmes EA Brown RJ Mansell W Fearon RP Hunter EC Frasquilho F . Are there two qualitatively distinct forms of dissociation? A review and some clinical implications. Clin Psychol Rev. (2005) 25:1–23. doi: 10.1016/j.cpr.2004.08.006. PMID: 15596078

[12] Bob P . Dissociation and neuroscience: history and new perspectives. Int J Neurosci. (2003) 113:903–14. doi: 10.1080/00207450390220376. PMID: 12881183

[13] American Psychiatric Association . Diagnostic and statistical manual of mental disorders: DSM-5. Washington, D.C: American Psychiatric Publishing (2013). doi: 10.1176/appi.books.9780890425596, PMID:

[14] World Health Organisation . International classification of diseases, eleventh revision (ICD-11) (2022). Available online at: https://icd.who.int/browse11 (Accessed January 22, 2026).

[15] Brown RJ Reuber M . Towards an integrative theory of psychogenic non-epileptic seizures (PNES). Clin Psychol Rev. (2016) 47:55–70. doi: 10.1016/j.cpr.2016.06.003. PMID: 27340856

[16] Campbell MC Smakowski A Rojas-Aguiluz M Goldstein LH Cardeña E Nicholson TR . Dissociation and its biological and clinical associations in functional neurological disorder: systematic review and meta-analysis. BJPsych. Open. (2023) 9:e2. doi: 10.1192/bjo.2022.597. PMID: 36451595 PMC9798224

[17] Yayla S Bakım B Tankaya O Ozer OA Karamustafalioglu O Ertekin H . Psychiatric comorbidity in patients with conversion disorder and prevalence of dissociative symptoms. J Trauma Dissociation. (2015) 16:29–38. doi: 10.1080/15299732.2014.938214. PMID: 25365395

[18] Scévola L Teitelbaum J Oddo S Centurión E Loidl CF Kochen S . Psychiatric disorders in patients with psychogenic nonepileptic seizures and drug-resistant epilepsy: a study of an Argentine population. Epilepsy. Behav. (2013) 29:155–60. doi: 10.1016/j.yebeh.2013.07.012. PMID: 23969203

[19] Kuyk J Siffels MC Bakvis P Swinkels WA . Psychological treatment of patients with psychogenic non-epileptic seizures: an outcome study. Seizure. (2008) 17:595–603. doi: 10.1016/j.seizure.2008.02.006. PMID: 18395473

[20] Mousa S Latchford G Weighall A Nash H Murray-Leslie R Reuber M . Evidence of objective sleep impairment in nonepileptic attack disorder: a naturalistic prospective controlled study using actigraphy and daily sleep diaries over six nights. Epilepsy. Behav. (2021) 117:107867. doi: 10.1016/j.yebeh.2021.107867. PMID: 33684785

[21] Akyüz F Gökalp PG Erdiman S Oflaz S Karşidağ Ç . Conversion disorder comorbidity and childhood trauma. Arch Neuropsychiatry. (2017) 54:15. doi: 10.5152/npa.2017.19184. PMID: 28566953 PMC5439465

[22] Millman LM Short E Ward E Stanton B Bradley-Westguard A Goldstein LH . Etiological factors and symptom triggers in functional motor symptoms and functional seizures: A pilot investigation. J Neuropsychiatry. Clin Neurosci. (2024) 36:350–7. doi: 10.1176/appi.neuropsych.20230103. PMID: 38481167

[23] Goldstein LH Mellers JD . Ictal symptoms of anxiety, avoidance behaviour, and dissociation in patients with dissociative seizures. J Neurol. Neurosurgery. Psychiatry. (2006) 77:616–21. doi: 10.1136/jnnp.2005.066878. PMID: 16614021 PMC2117432

[24] Brake B Wieder L Hughes N Lalinde IS Marr D Geagea D . The induction of dissociative states: A meta-analysis. Biol Psychiatry Global Open Sci. (2025) 5:100521. doi: 10.1016/j.bpsgos.2025.100521. PMID: 40530339 PMC12172952

[25] Caputo GB . Strange-face-in-the-mirror illusion. Perception. (2010) 39:1007–8. doi: 10.1068/p6466. PMID: 20842976

[26] Sierra M Berrios GE . Depersonalization: a conceptual history. History. Psychiatry. (1997) 8:213–29. doi: 10.1177/0957154X9700803002. PMID: 11619439

[27] Paredes-Echeverri S Maggio J Bègue I Pick S Nicholson TR Perez DL . Autonomic, endocrine, and inflammation profiles in functional neurological disorder: a systematic review and meta-analysis. J Neuropsychiatry. Clin Neurosci. (2022) 34:30–43. doi: 10.1176/appi.neuropsych.21010025. PMID: 34711069 PMC8813876

[28] Flasbeck V Jungilligens J Lemke I Beckers J Öztürk H Wellmer J . Heartbeat evoked potentials and autonomic arousal during dissociative seizures: insights from electrophysiology and neuroimaging. BMJ Neurol Open. (2024) 6:e000665. doi: 10.1136/bmjno-2024-000665. PMID: 38860229 PMC11163632

[29] Heekerens JB Schulze L Enge J Renneberg B Roepke S . Affective arousal temporally precedes dissociation in patients with borderline personality disorder: A preliminary experience sampling study. Psychol Trauma. (2024) 16:1129–38. doi: 10.1037/tra0001516. PMID: 37126047

[30] Pick S Millman LM Ward E Short E Stanton B Reinders AS . Unravelling the influence of affective stimulation on functional neurological symptoms: a pilot experiment examining potential mechanisms. J Neurol. Neurosurg Psychiatry. (2024) 95:461–70. doi: 10.1136/jnnp-2023-332364. PMID: 37963722 PMC11041613

[31] Krause-Utz A Frost R Winter D Elzinga BM . Dissociation and alterations in brain function and structure: implications for borderline personality disorder. Curr Psychiatry Rep. (2017) 19:6. doi: 10.1007/s11920-017-0757-y. PMID: 28138924 PMC5283511

[32] Wainipitapong S Millman LM Huang X Wieder L Terhune DB Pick S . Assessing dissociation: a systematic review and evaluation of existing measures. J Psychiatr Res. (2025) 181:91–8. doi: 10.1016/j.jpsychires.2024.11.040. PMID: 39603166 PMC11904123

[33] Kozlowska K Spooner CJ Palmer DM Harris A Korgaonkar MS Scher S . Motoring in idle The default mode and somatomotor networks are overactive in children and adolescents with functional neurological symptoms. NeuroImage.: Clin. (2018) 18:730–43. doi: 10.1016/j.nicl.2018.02.003. PMID: 29876262 PMC5987846

[34] Adewusi J Levita L Gray C Reuber M . Subjective versus objective measures of distress, arousal and symptom burden in patients with functional seizures and other functional neurological symptom disorder presentations: A systematic review. Epilepsy. Behav Rep. (2021) 16:100502. doi: 10.1016/j.ebr.2021.100502. PMID: 34917921 PMC8669370

[35] Wechsler D . Wechsler abbreviated scale of intelligence--second edition (WASI-II). APA. PsycTests. (2011) 31(3):337–41. doi: 10.1037/t15171-000. PMID: 41770175

[36] Pick S Millman LM Sun Y Short E Stanton B Winston JS . Objective and subjective neurocognitive functioning in functional motor symptoms and functional seizures: preliminary findings. J Clin Exp Neuropsychol. (2023) 45:970–87. doi: 10.1080/13803395.2023.2245110. PMID: 37724767 PMC11057846

[37] First MB Williams JBW Karg RS Spitzer RL . User’s guide for the structured clinical interview for DSM-5 disorders, research version (SCID-5-RV). Arlington, VA: American Psychiatric Association (2015).

[38] Millman LM Short E Stanton B Winston JS Nicholson TR Mehta MA . Interoception in functional motor symptoms and functional seizures: preliminary evidence of intact accuracy alongside reduced insight and altered sensibility. Behav Res Ther. (2023) 168:104379. doi: 10.1016/j.brat.2023.104379. PMID: 37516011 PMC10788481

[39] Pick S Millman LSM Davies J Hodsoll J Stanton B David AS . Real-time biopsychosocial antecedents and correlates of functional neurological symptoms in daily life: A pilot remote monitoring technology study. Psychiatry Res. (2024) 342:115424. doi: 10.1016/j.psychres.2024.116247. PMID: 39509765 PMC11876104

[40] Spitzer RL Kroenke K Williams JB Löwe B . A brief measure for assessing generalized anxiety disorder: the GAD-7. Arch Internal Med. (2006) 166:1092–7. doi: 10.1001/archinte.166.10.1092. PMID: 16717171

[41] Kroenke K Spitzer RL Williams JB . The PHQ-9: validity of a brief depression severity measure. J Gen Internal Med. (2001) 16:606–13. doi: 10.1046/j.1525-1497.2001.016009606.x. PMID: 11556941 PMC1495268

[42] Watson D Clark LA Tellegen A . Development and validation of brief measures of positive and negative affect: The PANAS scales. J Pers Soc Psychol. (1988) 54:1063–70. doi: 10.1037//0022-3514.54.6.1063. PMID: 3397865

[43] Bremner JD Krystal JH Putnam FW Southwick SM Marmar C Charney DS . Measurement of dissociative states with the Clinician-Administered Dissociative States Scale (CADSS). J Traumatic. Stress. (1998) 11:125–36. doi: 10.1023/A:1024465317902. PMID: 9479681

[44] Hox J Moerbeek M van de Schoot R . Multilevel analysis: techniques and applications, third edition. New York: Routledge (2017). doi: 10.4324/9781315650982

[45] Hox JJ Maas CJ . Sample sizes for multilevel modeling. (2002) 1(3):86–92. doi: 10.1027/1614-2241.1.3.85

[46] Pick S David AS Edwards MJ Goldstein LH Hodsoll J Millman LSM . Investigating psychobiological causes and mechanisms in functional seizures and functional motor symptoms: Study protocol. PloS One. (2024) 19:e0305015. doi: 10.1371/journal.pone.0305015. PMID: 38905248 PMC11192335

[47] Beutler-Traktovenko S Franz M Daniels J Schellong J Weidner K Croy I . Dissociative episodes and concurrent heart-rate in patients with PTSD – An ecological momentary assessment. Psychiatry Res. (2025) 344:116345. doi: 10.1016/j.psychres.2024.116345. PMID: 39798482

[48] Nicholson TR Aybek S Craig T Harris T Wojcik W David AS . Life events and escape in conversion disorder. Psychol Med. (2016) 46:2617–26. doi: 10.1017/S0033291716000714. PMID: 27377290 PMC4988265

[49] Bakvis P Roelofs K Kuyk J Edelbroek PM Swinkels WA Spinhoven P . Trauma, stress, and preconscious threat processing in patients with psychogenic nonepileptic seizures. Epilepsia. (2009) 50:1001–11. doi: 10.1111/j.1528-1167.2008.01862.x. PMID: 19170739

[50] Ludwig L Pasman JA Nicholson T Aybek S David AS Tuck S . Stressful life events and maltreatment in conversion (functional neurological) disorder: systematic review and meta-analysis of case-control studies. Lancet Psychiatry. (2018) 5:307–20. doi: 10.1016/s2215-0366(18)30051-8. PMID: 29526521

[51] Pick S Mellers JD Goldstein LH . Autonomic and subjective responsivity to emotional images in people with dissociative seizures. J Neuropsychol. (2018) 12:341–55. doi: 10.1111/jnp.12144. PMID: 29285879 PMC6001553

[52] Herrero H Tarrada A Haffen E Mignot T Sense C Schwan R . Skin conductance response and emotional response in women with psychogenic non-epileptic seizures. Seizure. (2020) 81:123–31. doi: 10.1016/j.seizure.2020.07.028. PMID: 32795943

[53] Pick S Goldstein LH Perez DL Nicholson TR . Emotional processing in functional neurological disorder: a review, biopsychosocial model and research agenda. J Neurol. Neurosurg Psychiatry. (2019) 90:704–11. doi: 10.1136/jnnp-2018-319201. PMID: 30455406 PMC6525039

[54] McLoughlin C Hoeritzauer I Cabreira V Aybek S Adams C Alty J . Functional neurological disorder is a feminist issue. J Neurol. Neurosurg Psychiatry. (2023) 94:855–62. doi: 10.1136/jnnp-2022-330192. PMID: 36977553 PMC10511956

[55] Lyssenko L Schmahl C Bockhacker L Vonderlin R Bohus M Kleindienst N . Dissociation in psychiatric disorders: A meta-analysis of studies using the dissociative experiences scale. Am J Psychiatry. (2018) 175:37–46. doi: 10.1176/appi.ajp.2017.17010025. PMID: 28946763

