# Supplementary material for: Momentary predictors of dissociation in functional neurological disorder: an ecological momentary assessment-based pilot study
Source: Front Psychiatry. 2026 Apr 30;17:1798482. doi: 10.3389/fpsyt.2026.1798482 (PMC13171792; doi:10.3389/fpsyt.2026.1798482)
Supplement: Supplementary file 1 [file Table1.docx]

**Supplementary Materials**

**S1 – Study design and Analytical Framework**

Data were hierarchically structured with repeated observations (Level 1; n = 56) nested within individuals (Level 2; n = 17 in each group). Linear multilevel models were estimated using maximum likelihood (ML) with Satterthwaite approximation for degrees of freedom. A random intercept for participant was specified with a variance components covariance structure. Random slopes were not included.

**S2 – Descriptive Statistics**

Table S1 – Descriptive Statistics for all variables

|  | **Amnesia** | **Depersonalisation** | **Derealisation** | **Heart Rate** | **Arousal** | **Negative affect** | **GAD-7** | **PHQ-9** |
| --- | --- | --- | --- | --- | --- | --- | --- | --- |
| **N (Valid)** | 1517 | 1516 | 1515 | 1207 | 1524 | 1522 | 1904 | 1904 |
| **N (Missing)** | 387 | 388 | 389 | 697 | 380 | 382 | 0 | 0 |
| **Mean** | 1.4841 | 1.4555 | 1.4809 | 0.2166 | -0.0001 | -0.0006 | 5.8556 | 7.2952 |
| **SD** | 0.96921 | 1.13240 | 1.09715 | 11.27331 | 0.98992 | 0.46646 | 5.27675 | 6.42537 |

*NB: Heart Rate, Arousal, and Negative affect represent person-centred variables. Statistics reflect the full dataset prior to multilevel decomposition.*

Table S2: Questions and scoring from ecological momentary assessment questionnaire as presented in Pick *et al.* (2024a) license CC-BY4.

| Domain | Question and scale for scoring (1 = Not at all – 7 = Extremely): |
| --- | --- |
| Dissociation: Dissociative amnesia | a) I cannot account for things that have recently happened.  b) I feel spaced out, and/or have lost track of what is going on. |
| Dissociation: Depersonalisation | a) I feel disconnected from my own body.  b) I feel separated from what is happening to me, like an actor in a movie, or a robot. |
| Dissociation: Derealisation | a) Things seem unreal to me, as if I am in a dream.  b) It seems like I am looking at the world through a fog. |
| Negative affect | I feel:   - Scared - Upset - Nervous - Ashamed - Irritable - Hostile |
| Arousal | I feel bodily arousal. * |

**Participants were instructed to report on bodily arousal reflecting markers of sympathetic/autonomic arousal, with examples provided (e.g. racing heart, sweating, dry mouth).*

**S3 – Intraclass Correlations**

Table S3 – Intraclass Correlation Coefficients (ICCs)

| **Outcome** | **Unconditional ICC** | **Adjusted ICC** |
| --- | --- | --- |
| Amnesia | .57 | .53 |
| Depersonalisation | .79 | .63 |
| Derealisation | .81 | .61 |

**S4 – Between-Person Correlations**

Table S3 – Between-Person Correlations (Pearson’s *r)*

| **Variable** | **1** | **2** | **3** | **4** | **5** |
| --- | --- | --- | --- | --- | --- |
| 1. Amnesia | — |  |  |  |  |
| 2. Depersonalisation | .60–.78 | — |  |  |  |
| 3. Derealisation | .53–.79 | .88–.94 | — |  |  |
| 4. PHQ-9 | .02–.54 | .20–.33 | .28–.32 | — |  |
| 5. GAD-7 | .25–.32 | .23–.34 | .12–.25 | .64–.79 | — |

*NB. Values represent Pearson correlations. Ranges reflect FND–HC values.*

**S5 Within-Person Correlations**

Table S4 – Within-Person Correlations (Person-mean centred variables)

| **Variable** | **1** | **2** | **3** | **4** | **5** | **6** |
| --- | --- | --- | --- | --- | --- | --- |
| 1. HR | — |  |  |  |  |  |
| 2. Arousal | .05 | — |  |  |  |  |
| 3. Negative Affect | -.02 | .32 | — |  |  |  |
| 4. Amnesia | .01 | .21 | .19 | — |  |  |
| 5. Depersonalisation | -.02 | .17 | .16 | .54 | — |  |
| 6. Derealisation | -.00 | .20 | .20 | .49 | .58 | — |

**S6 – Multilevel Model Equations**

**Step One: Single predictor Models**

**Model One: Arousal only**

Level 1: Y_ti = β_0i + β_1(Arousal_ti − Arousal̄_i) + e_ti

Level 2: β_0i = γ_00 + u_0i

**Model Two: Negative Affect Only**

Level 1: Y_ti = β_0i + β_1(NegAff_ti − NegAff̄_i) + e_ti

Level 2: β_0i = γ_00 + u_0i

**Model Three: Heart Rate only**

Level 1: Y_ti = β_0i + β_1(HR_ti − HR̄_i) + e_ti

Level 2: β_0i = γ_00 + u_0i

**Step Two: Full combined model and sensitivity analyses**

**Level 1 (Within-Person):**

Y_ti = β_0i + β_1(Arousal_ti − Arousal̄_i) + β_2(NegAff_ti − NegAff̄_i) + β_3(HR_ti − HR̄_i) + e_ti

**Level 2 (Between-Person):**

β_0i = γ_00 + γ_01(Group_i) + γ_02(PHQ9_i) + γ_03(GAD7_i) + u_0i

**S7 – Variance Structure**

A variance components covariance structure was specified:

- Random intercept variance estimated at Level 2
- Residual variance estimated at Level 1
- No covariance parameters among slopes (no random slopes specified)

**S8 – Model Building Strategy**

Models were estimated sequentially:

1. Unconditional random intercept model (ICC estimation)
2. Addition of Level 1 predictors
3. Addition of between-person predictors (Group, PHQ-9, GAD-7)

Model fit was evaluated using −2LL, AIC, and BIC under ML estimation.

**S9 – Details on Ecological Momentary Assessment (EMA) Variable Constructs**

In the development of the CADSS and PANAS, as reported by Bremner et al. (1998) and Watson et al. (1988), both scales have shown high internal consistency, validity, and reliability. Cronbach’s alpha were calculated for the negative affect items (α=.78), depersonalisation (α=.90), derealisation (α=.81), amnesia (α=.64), and total dissociation items (α=.91) that were included in EMA prompts in this study.

Variance inflation factors (VIF) were examined to assess multicollinearity between negative affect and subjective arousal. The VIF values were low (1.18), suggestive of no evidence of problematic multicollinearity.

**S10 – Details on missing data**

Two FND participants did not record Heartrate data, one due to device loss and one due to syncing issues. One Healthy Control participant did not wear the FitBit 5 device, hence data was not collected. This was in addition to some heart rate data which was missing at random from other participants who did use the device. This resulted in higher levels of missing heart rate data when contrasted with other variables. Missing data was not imputed, and as multilevel models are suitable for use with missing data, a cut-off for exclusion was not specified.

The other variables all had ≥80% response to prompts.
